# *SLC34A3* Intronic Deletion in an Iranian Kindred with Hereditary Hypophosphatemic Rickets with Hypercalciuria

**DOI:** 10.4274/jcrpe.0057

**Published:** 2018-11-29

**Authors:** Shirin Hasani-Ranjbar, Hanieh-Sadat Ejtahed, Mahsa M. Amoli, Fatemeh Bitarafan, Mostafa Qorbani, Akbar Soltani, Bahareh Yarjoo

**Affiliations:** 1 Obesity and Eating Habits Research Center, Endocrinology and Metabolism Clinical Sciences Institute, Tehran University of Medical Sciences, Tehran, Iran; 2 Endocrinology and Metabolism Research Center, Endocrinology and Metabolism Clinical Sciences Institute, Tehran University of Medical Sciences, Tehran, Iran; 3 Metabolic Disorders Research Center, Endocrinology and Metabolism Molecular-Cellular Sciences Institute, Tehran University of Medical Sciences, Tehran, Iran; 4 Non-communicable Diseases Research Center, Alborz University of Medical Sciences, Karaj, Iran; 5 Evidence-Based Medicine Research Center, Endocrinology and Metabolism Clinical Sciences Institute, Tehran University of Medical Sciences, Tehran, Iran

**Keywords:** Hereditary hypophosphatemic rickets with hypercalciuria, SLC34A3 gene, hypophosphatemia, hypercalciuria

## Abstract

**Objective:**

To describe clinical findings, biochemical profile and genetic analysis in an Iranian kindred with hereditary hypophosphatemic rickets with hypercalciuria (HHRH).

**Methods:**

Clinical examination and biochemical profile results and gene analysis of 12 members of a family of a patient previously diagnosed with HHRH due to *SLC34A3* mutation. Ten healthy controls were also evaluated.

**Results:**

Of the twelve family members three were homozygote and seven heterozygote for the same *SLC34A3* variant found in the proband while two others were unaffected. All patients had significantly increased risk of kidney stone formation, bone deformities and short stature compared with unrelated healthy controls. The heterozygous patients displayed milder clinical symptoms compared with homozygous patients. In particular they had mild or no hypophosphatemia and they did not develop skeletal deformities. Recurrent renal stones and hypercalciuria were the main presentations of the heterozygous patients which may be confused with familial hypercalciuria. In addition, biochemical analysis showed significantly low serum sodium and elevated alkaline phosphatase levels in these patients.

**Conclusion:**

Genetic counseling and screening for *SLC34A3* mutations can be helpful in adult onset phenotype with unexplained osteoporosis, bone deformities and especial recurrent renal stones. In subjects with vitamin D deficiency the results should be interpreted cautiously.

**What is already known on this topic?**

Hereditary hypophosphatemic rickets with hypercalciuria is a very rare inheritable hypophosphatemic rickets/osteomalacia. Biallelic mutations in the *SLC34A3/NPT2c* gene are responsible for the disease.

**What this study adds?**

In this paper, we describe clinical findings, biochemical profile and gene analysis of Iranian kindred with a 101bp deletion in the *SLC34A3* gene. Genetic counseling and screening for *SLC34A3* mutations can be helpful in adult onset phenotype with unexplained osteoporosis, bone deformities and recurrent renal calculi.

## Introduction

Loss of function in the third member of the sodium phosphate cotransporter family type II (NaPi-IIc/*NPT2c*) which is encoded by the *SLC34A3* gene causes hereditary hypophosphatemic rickets with hypercalciuria (HHRH) (1). HHRH is a rare metabolic disorder (OMIM #241530) with an autosomal recessive mode of inheritance that was first described in a large, consanguineous Bedouin kindred (1,2,3). The candidate gene, which is located on chromosome 9q34, codes for NaPi-IIc/*NPT2c* which is expressed at the apical domain of renal proximal tubule cells and plays a fundamental role in the maintenance of phosphate homeostasis (2,4). *NPT2c* contributes to renal phosphate reabsorption from glomerular filtrate under the hormonal control of parathyroid hormone (PTH) and fibroblast growth factor 23 (2). Phosphate participates in a remarkably wide array of cellular processes, intracellular signaling, pH buffering, bone mineralization, phospholipid structures and nucleic acids synthesis (4). Clinically, HHRH patients, who carry homozygous or compound heterozygous *SLC34A3/NPT2c* mutations, often show hypophosphatemia following decreased renal phosphate reabsorption, rickets and/or osteomalacia and frequently kidney stones or nephrocalcinosis. Hypophosphatemia is followed by upregulation of renal 1-a-hydroxylase and increased serum level of 1.25-dihydroxy vitamin D (1.25(OH)_2_D) resulting in elevated intestinal absorption of calcium and urinary calcium excretion despite suppressed parathyroid function (1,5,6). High serum 1.25(OH)_2_D concentrations and hypercalciuria distinguish HHRH from other hypophosphatemic disorders (7). Other features include slow growth, limb deformities, muscle weakness, bone pain, bowing and short stature (5,8,9). Since the initial description a few families and sporadic cases have been reported in Turkey, Holland, Morocco, North America, Japan, Africa, Caucasus, Germany and Iran (2,9,10,11). This study reports a case series in a kindred describing clinical features, biochemical profile and subsequent candidate gene analysis of a family with a 101-bp intronic deletion within the *SLC34A3* gene.

## Methods

**Figure 1:**
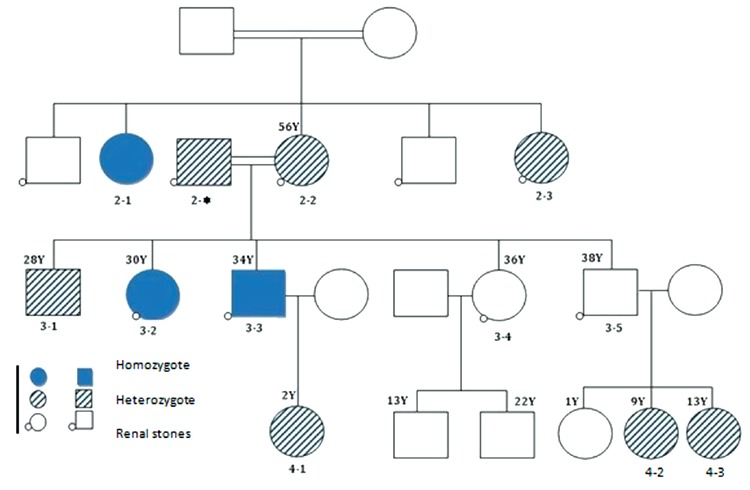
Genetic relationship of patients with hereditary hypophosphatemic rickets with hypercalciuria. Genetic analysis was not done for subject 3-5

Twelve members of a family of a previously reported patient (11) with HHRH (Figure 1) were evaluated in the endocrine unit of the Shariati Hospital, Tehran University of Medical Sciences. Analysis of the extended family’s medical history disclosed other members with a history of nephrolithiasis. Ten unrelated healthy subjects (no deformity, no history of renal calculi or calcium and bone disease) were included in this study as the control group. The study was approved by the Ethical Committee of Endocrinology and Metabolism Research Institute, Tehran University of Medical Sciences (No: IR.TUMS.EMRI.REC.1390). Written informed consent was obtained from the family.

A detailed clinical examination was conducted to identify any physical signs and symptoms of rickets including; skeletal deformities, leg pain, difficulty in walking, and leg bowing. Height and weight were measured in all patients. Skeletal X-rays were taken and renal ultrasonography and radiological examination were performed. Bone mineral density (BMD) of the spine, right hip and forearm was assessed for two of the homozygous patients using dual-energy X-ray absorptiometry.

Twelve hour overnight fasting blood and 24 hour urine samples were collected to measure calcium, creatinine and phosphorus. Serum levels of intact PTH, serum 25-hydroxyvitamin D [25(OH)D] and total alkaline phosphatase were assessed. Fasting tubular reabsorption of phosphate (TRP) and maximal renal phosphate reabsorption per glomerular filtration rate (TMP/GFR) were calculated using the following formula: 1 - (urine phosphorus × serum creatinine/serum phosphorus × urine creatinine).

### Genetic Analysis

Genomic DNA was isolated from peripheral blood leukocytes. The previously detected 101bp deletion in intron 9 of the *SLC34A3* gene was screened for after direct sequencing of the entire *SLC34A3* gene on ABI 3130 genetic analyzer (Applied Biosystems, Thermo Fisher Scientific corporation, USA) as described by Bergwitz et al (8).

### Statistical Analysis

All statistical analysis was performed using SPSS software version 16 (IBM Inc., Chicago, Ill., USA). Normal distribution of continuous variables was assessed using Kolmogorov-Smirnov test. Continuous variables with normal distribution are presented as mean (standard deviation). Comparison of continuous variables between groups was done using ANOVA test. P values <0.05 were considered as statistically significant.

## Results

A total of 12 individual members of an HHRH kindred were included in this study, three of whom were homozygote and seven heterozygote whilst the remaining two were healthy. Genetic analysis revealed the presence of a previously detected 101-bp deletion in intron 9 (Figure 1). BMD of the spine, right hip and forearm in two homozygous patients was measured (Table 1) and very low BMD and osteoporosis was found (one of homozygous patient did not consent to do the BMD). The T score of the spine, right hip and forearm were -1.6 and -3.4, -2.7 and -2.3, -3.9 and -3.7 in patients 3-3 and 3-2, respectively. Table 2 shows clinical examination and biochemistry results carried out on the homozygous and heterozygous individuals of the investigated kindred. Seven members of the kindred had a history of kidney stone. Serum creatinine was high in two members (3-1 and 3-3) and serum calcium was high in another two members (4-1 and 4-3). In six of the eight adults tested alkaline phosphatase level was found to be above the reference range. Comparison of biochemical examinations between mutant homozygous and heterozygous individuals in the kindred and healthy controls was shown in Table 3. Serum concentrations of sodium and alkaline phosphatase and mean corpuscular hemoglobin concentration were significantly different among the three groups (p<0.05). Serum concentrations of sodium, potassium and calcium were lower in homozygous individuals compared to normal individuals. Serum alkaline phosphatase was higher in homozygous and heterozygous individuals in comparison with healthy controls (p=0.008). Elevated hematocrit and low serum phosphate levels were not significantly different. Twenty four hour urine volume and urine calcium were higher in homozygous patients.

Homozygote and heterozygote mutations in the *SLC34A3* gene were found to lead to a significantly increased risk of kidney stone formation and bone deformities which in turn led to short stature and growth delay.

## Discussion

In this case series study, we describe and discuss the clinical examination, biochemical profile and gene analysis results of the family members (affected and unaffected) of an HHRH patient. A total of 12 individuals of an Iranian kindred were included in this study. Of these, three were homozygote, seven heterozygote and two were healthy.

Homozygote and heterozygote mutations in the *SLC34A3* gene lead to a significantly increased risk of kidney stone formation and bone deformities, compared with healthy controls. A hallmark feature of familial hypophosphatemic rickets is short stature resulting from deformity and growth retardation, which is observed in both homozygous and heterozygous individuals. Hypophosphatemia in HHRH leading to elevation in the serum level of 1.25(OH)_2_D which results in hypercalciuria. Episodes of hypercalciuria may cause development of recurrent renal stones. Based on biochemical follow-up data that were available for the homozygote and heterozygote members in this kindred, significantly low serum sodium levels and elevated alkaline phosphatase levels were observed.

The causative gene, *SLC34A3*, which is mapped to chromosome 9q34 encodes a member of the SLC34A transporter family of proteins which is involved in transporting phosphate into cells mediated by sodium-phosphate cotransporters in the renal brush border membrane and plays a key role in phosphate homeostasis, despite its low expression levels (4,8). It has been demonstrated that mice homozygous for the disrupted *NPT2* gene show many of the features of HHRH and that the *SLC34A1/NaPi7* gene plays a key role in phosphate homeostasis and in normal skeletal development (12). Not many cases of biallelic *SLC34A3/NPT2c* mutations resulting in HHRH syndrome, have been reported worldwide (5). Different mutations in *SLC34A3/NPT2c* with different phenotypes have been reported in these patients (1,2,5,6,8,9,10,13,14,15,16,17). This present study reports the first kindred of HHRH in Iran and describes a previously described mutation, a 101bp deletion, within the *SLC34A3* gene, which affects transcription or splicing of pre-mRNA, causes aberrant RNA splicing, between exons 9 and 10 (11).

Since HHRH is an autosomal recessive disease, biallelic mutations are required for full-scale disease manifestations; loss of one *SLC34A3* allele does not always lead to laboratory abnormalities. However, clinical phenotypes are sometimes seen in carriers of single *SLC34A3* mutations (2,6). Similarly, several heterozygous members of the Bedouin kindred for the c.228delC mutation, displayed mild hypophosphatemia, reduced TMP/GFR, and elevations in 1.25(OH)_2_D concentrations in addition to increased urinary calcium excretion (8). In the present study, the heterozygous patients displayed milder clinical symptoms compared with homozygous patients. These patients displayed mild or no hypophosphatemia and they did not develop skeletal deformities. Recurrent renal stones and hypercalciuria were the main presenting features of these patients, which could be confused with familial hypercalciuria. HHRH diagnosis can be missed in this situation and appropriate interpretation of the clinical symptoms is important. Absence of clinical symptoms and of biochemical alterations have also been reported in previous heterozygous HHRH families (2,5,6,8,17). Therefore, genetic screening for *SLC34A3* mutations can be helpful in patients with suspicious clinical findings.

Hypophosphatemia and renal stones are common in homozygous patients. In addition bone deformities may not always develop in these patients and may create difficulties in diagnosing this disease. Further investigation and genetic evaluation are needed in this situation.

Poor vitamin D status is highly prevalent among Iranian adults with vitamin D deficiency and insufficiency reported in 90.7% of the adult population (18). Moreover, hypercalciuria and renal stones are prevalent in Iran from childhood, as patient 3-4 also had kidney stones without any mutation (19,20). Thus, evaluation of vitamin D level and/or increase in serum creatinine should also be measured when assessing serum and urine levels of phosphorous and calcium. As the findings of patients 3-1 and 3-3 demonstrate, chronic kidney diseases and vitamin D deficiency are two important issues for interpretation of biochemical findings.

### Study Strengths and Study Limitations

The large number of individuals included in the study is the strength of this study. However, we realize that the sample size of the control cohort is small. One of the limitations of the study was the lack of clinical and genetic testing for all members of the family. Moreover, the concentration of 1.25(OH)_2_D_3_ which is very important in differentiating HHRH patients, was not measured in this study. It should be noted that the 24 hour urine samples are influenced by dietary phosphate and three hour, fasting, spot urines were not determined in order to calculate TRP%. In addition, genes encoding other phosphate transporters were not sequenced.

## Conclusion

In conclusion, as the clinical phenotype of HHRH can be quite variable with different penetrance even in the same family with identical mutations, it is not possible to be certain about a genotype-phenotype effect, and a proper diagnosis requires molecular genetic analysis. Screening for *SLC34A3* mutations to evaluate the importance of treatment and close follow-up to avoid complications can be helpful.

## Figures and Tables

**Table 1:**
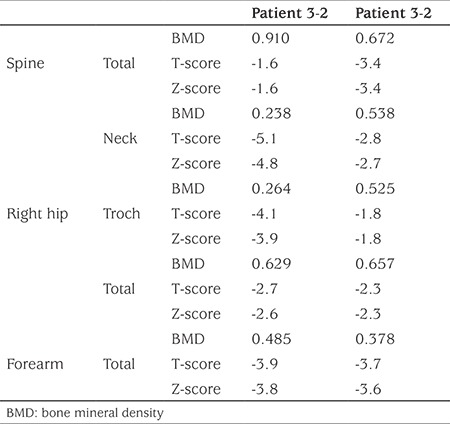
Comparison of bone mineral densitometry values (g/cm^2^) of spine, right hip and forearm in two of the homozygous patients

**Table 2:**
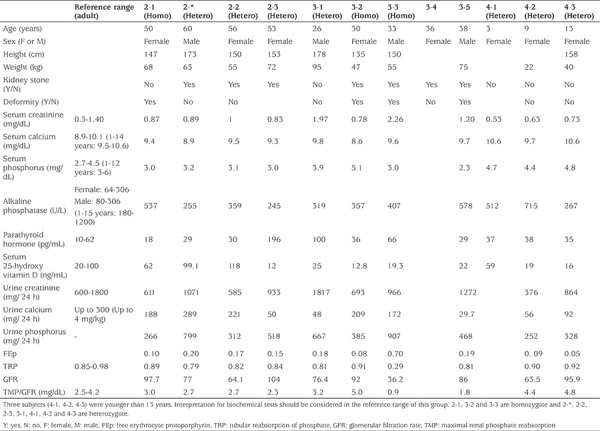
Clinical examination and biochemical findings of the mutant homozygous and heterozygous individuals

**Table 3:**
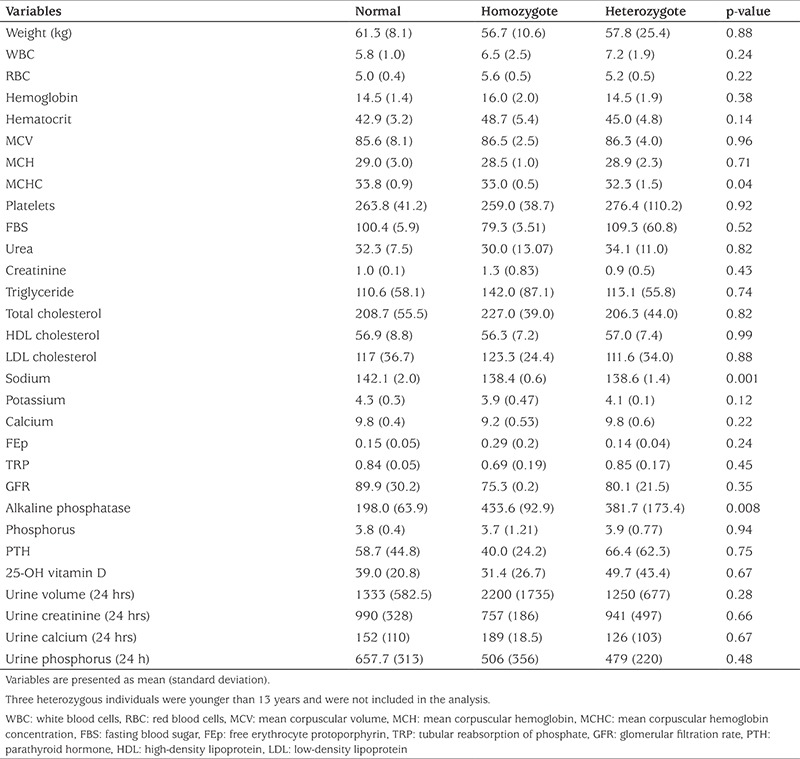
Comparison of biochemical findings between mutant homozygote and heterozygote and normal controls
